# Periodontal health in a large cohort of Ugandansliving with HIV: A cross-sectional study

**DOI:** 10.21203/rs.3.rs-4555317/v1

**Published:** 2024-06-28

**Authors:** Buwembo William, Kamulegeya Adriane, Kalanzi Dunstan, Namuyonga Priscilla Naava, Nakasujja Proscovia, P Katete David, Semitala F. Collins, Mwesigwa-Lutalo Catherine, Kalungi Samuel, Cameron Jennnifer E, Munabi Ian G

**Affiliations:** Makerere University College of Health Sciences; Makerere University College of Health Sciences; Makerere University College of Health Sciences; Makerere University College of Health Sciences; Makerere University College of Health Sciences; Makerere University College of Health Sciences; Makerere University College of Health Sciences; Makerere University College of Health Sciences; Mulago National Referral and Teaching Hospital; Louisiana State University Health Sciences Center: New Orleans; Makerere University College of Health Sciences

**Keywords:** Periodontal health, Periodontitis, Aging, HIV, HAART

## Abstract

**Background:**

The impact of periodontitis on large populations of people living with HIV (PLHIV) in resource-constrained settings remains largely un-investigated. This study aims to address this knowledge gap by providing a comprehensive description of the periodontal health status among a sizable cohort of Ugandans living with HIV.

**Methods:**

This was a cross-sectional study with 4,449 participants who were over 18-years old and captured their reported age, gender, tobacco use, length of time on HAART and alcohol use. Periodontal health was assessed using the WHO periodontal probe and the modified CPI data entry form. Descriptive statistics were reported using frequencies for the affected number of sextants in the surveyed participants. This was followed by additional regression analysis using the R statistical computing environment, with the periodontal health outcomes (bleeding on probing, pocket depth and clinical attachment loss) individually as the dependant, recoded as binary outcomes. A multilevel model was run with clinical attachment loss as the dependant variable controlling for all the other factors. The 95% confidence intervals were used to report the level of significance for each test.

**Results:**

There were 3,103/4,449 (69.7%) female participants. The mean age was 44.3 years (SD 10.1 years) with a range of 18 to 89 years. About 66% of the participants had bleeding on probing at one or more of the examined sites/tooth surfaces. The odds for bleeding on probing were significantly higher for female participants (adjusted Odds ratio: 1.49, 95% CI 1.19 to 1.86), and higher in individuals who reported tobacco use (adjusted odds ratio 1.62, 95% CI 1.09 to 2.41). Slightly under half of our participants (48.2%) had moderate to severe clinical attachment loss.

**Conclusions:**

This study found that among Ugandans living with HIV, periodontal disease is a significant public health concern. The majority (66%) had bleeding on probing, with a sizeable number (48.2%) of participants recording moderate to severe clinical attachment loss, worsened by age and time on HAART. This highlight the need for comprehensive oral health care and targeted interventions for this population.

## Background

Sub-Saharan Africa, the region most affected by HIV globally, faces a growing challenge: the number of people over 50 with HIV is expected to double by 2050 compared to 2020 levels [1]. This is due to the success of antiretroviral therapy’s (ARTs), allowing people with HIV to live longer, but also because sub-Saharan Africa carries 69% of the world’s HIV burden. Although longevity is a blessing, it comes with a greater burden of aging related conditions. Unfortunately, some these affect the patient’s ability to carry out oral hygiene interventions hence affecting overall oral health [2]. Unfortunately, aging associated conditions such as neurocognitive impairment and frailty have a direct effect of one’s ability to carry out routine oral hygiene measures [3, 4].

There is also emerging evidence of bidirectional effects between periodontal health, frailty and neurocognitive decline [5–7]. Periodontitis, which is defined as a chronic inflammatory disease of infectious origin, manifests as destruction of the tissue supporting the teeth [8]. Periodontitis has also been described as a group of multifactorial, inflammatory infectious diseases, characterised by an ecological shift in the composition of sub gingival biofilm, with an aberrant host response and inflammatory destruction of tooth-supporting tissues, all eventually leading to tooth loss [9, 10]. The severe forms of periodontitis currently affect approximately 19% of the global adult population, that is about 1 billion cases worldwide [11].

The severity of periodontitis is associated with increased prevalence of various periodonto-pathogenic bacteria and inflammation. This inflammation combined with other factors related to HIV infection contribute to accelerating periodontal disease progression [12]. Periodontitis has also been associated or identified as one of the risk factors in several medical conditions including: rheumatoid arthritis [13, 14], frailty [15], and dementia [16]. For most of these conditions the link between periodontitis and the above mentioned diseases are the pro-inflammatory agents such as IL-1β, TNF-α, and IL-6 [17]. Periodontitis is also exacerbated by tobacco use usually in the form of smoking and alcohol consumption. Tobacco specifically impairs the cellular level self-repair processes in the oral mucosa which increases cellular senescence and eventually inflammation [18]. PLHIV are more likely to develop the moderate to severe forms of periodontitis compared with the non-HIV general population [19, 20]. Also, increasing age is positively associated with periodontitis in both PLHIV and those without HIV. A combination of HIV and aging is likely to enhance periodontal challenges [19, 21, 22]. In this resource limited setting the impact of periodontitis on a large cohort of PLHIV [23], remains largely undocumented. In this study we set out to describe the periodontal health of a large cohort of Ugandans living with HIV.

## Methods

This was a cross-sectional study carried out at the large Makerere Joint Aids Program (MJAP) urban HIV clinic that currently supports the care for a total of 14,000 Ugandan people living with HIV (PLHIV) [24]. The MJAP clinic is in Kampala, the capital city of Uganda, next to the Makerere University College of Health Sciences. Participants were recruited from this catchment population into the study between October 2022 to October 2023. The data presented in this manuscript was collected as a sub study under a larger ongoing study looking at oral human papilloma virus, microbiota, and cancer in People Living with HIV. The inclusion criteria for participants were that one had to be above the age of 18-years and registered to receive treatment at the clinic. In addition, one had to have come for their scheduled drug refills as evidenced by their prescription for that day to be admitted into the study. We excluded participants with a history of having undergone any form of periodontal treatment. The data and research participants who were examined during the training of the clinical examiners were both excluded from the main study.

Variables: these included participants’ reported age, gender, tobacco use, background information on oral care practices, how long they had been on HAART and alcohol use. Each participant had a dental examination by a qualified degree holding calibrated dental surgeon supported by a dental research assistant that assessed for: registration of plaque (defined as being present or absent at six points on each present mature completely erupted sentinel teeth in each sextant), bleeding on probing (recorded as either present or absent within 30 seconds of probing), probing depth (these measurements in millimeters were performed on selected teeth in each sextant, on six sites per tooth using a manual WHO periodontal probe (Ball size 0.5mm) MEDENTRA ^™^, UK) and the number of teeth found on examination.

Sample size: for this sub study was calculated using the online sample size calculator [25], for sample sizes based on proportions, with the following assumptions: total population of 80,000, alpha of 0.05, power (1-beta) of 0.95, prevalence of periodontitis in people living with HIV from another sub-Saharan African country 34.7% [26], precision of 2% and design effect of 2 to cater for the multiple measurements within the same person. This gave a final sample size of 4,237. To this was added a 5% allowance for errors and omissions to give a final sample size of 4,449 participants.

### Training of examiners

The dental surgeons and research (dental) assistants received a weeks’ worth of training in how to assess periodontal health using the WHO probe and the modified CPI data entry form. In both the training and the later clinical data collection the instructions in the World Health Organisation (WHO) manual, were followed, with each dental examination focused on a tooth from each sextant in the upper and lower jaw of the mouth to align the data obtained with other epidemiological surveys [27]. The training involved an initial discussion of the research protocol and the data collection tools that were used for the study. This was followed by hands-on training that consisted of a series of three supervised examinations by each Team (Dental surgeon and research (dental) assistant) and an experienced clinician who was the reference. For each examination measurements were made of the bleeding on probing, pocket depth and attachment loss were captured by the team (dental surgeon and research dental assistant) and the experienced clinician following the guidelines in the WHO manual. The average inter-rater level of agreement was 0.71, while the mean repeated self-rater level of agreement as 0.85.

### Participant recruitment and Data collection

To ease participation, the site of recruitment was located on the same premises as the HIV clinic to minimize long distance movements and costs. Participants were recruited consecutively on the respective days of their scheduled visits for drug refills. The identified participants provided the study team with their informed consent to participate in the study. Those who agreed to enroll into the study proceeded to complete the study questionnaire, which was followed by the periodontal examination of the selected teeth in each sextant, following the instructions in the World Health Organisation (WHO) manual for each participant’s six sentinel/Ramfjord teeth [27]. Data was captured digitally in real time using CRFs in the Redcap app on a tablet computer.

### Statistical methods Analysis

The focus of the analysis was on the comparison of the participants periodontal health and their reported age. The reporting of the results followed the recommendation of the 2013 WHO modified CPI Oral health Assessment form for adults [27]. According to this manual, descriptive statistics are reported using frequencies for the affected number of sextants in the surveyed participants. This was followed by additional regression analysis using the glmmTMB package [28], in the R statistical computing environment version 4.3.1[25], with the periodontal health outcomes (bleeding on probing, pocket depth and clinical attachment loss) individually as the dependant variables. These were re-coded as binary outcomes prior to analysis. A backwards stepwise regression modelling strategy was used to identify a final model with the lowest AIC and only significant predictors for each outcome. Finally, a multilevel model was run with tooth level clinical attachment loss as the dependant variable controlling for all the other factors. We used the 95% confidence intervals to report the level of significance for each statistical computation, using only the complete observations (i.e., complete case analysis).

#### Ethical approval:

The parent study, titled “Oral Papilloma Virus, Microbiota and Cancer in People Living with HIV (OHPVMC)”, was approved by the Makerere University School of Medicine Research and Ethics committee SOMREC (REC REF 2022–451) and the Uganda National Council of Science and technology (HS2541ES). All participants gave their written informed consent to participate in all aspects of the approved study prior to recruitment.

## Results

**Figure 1:**
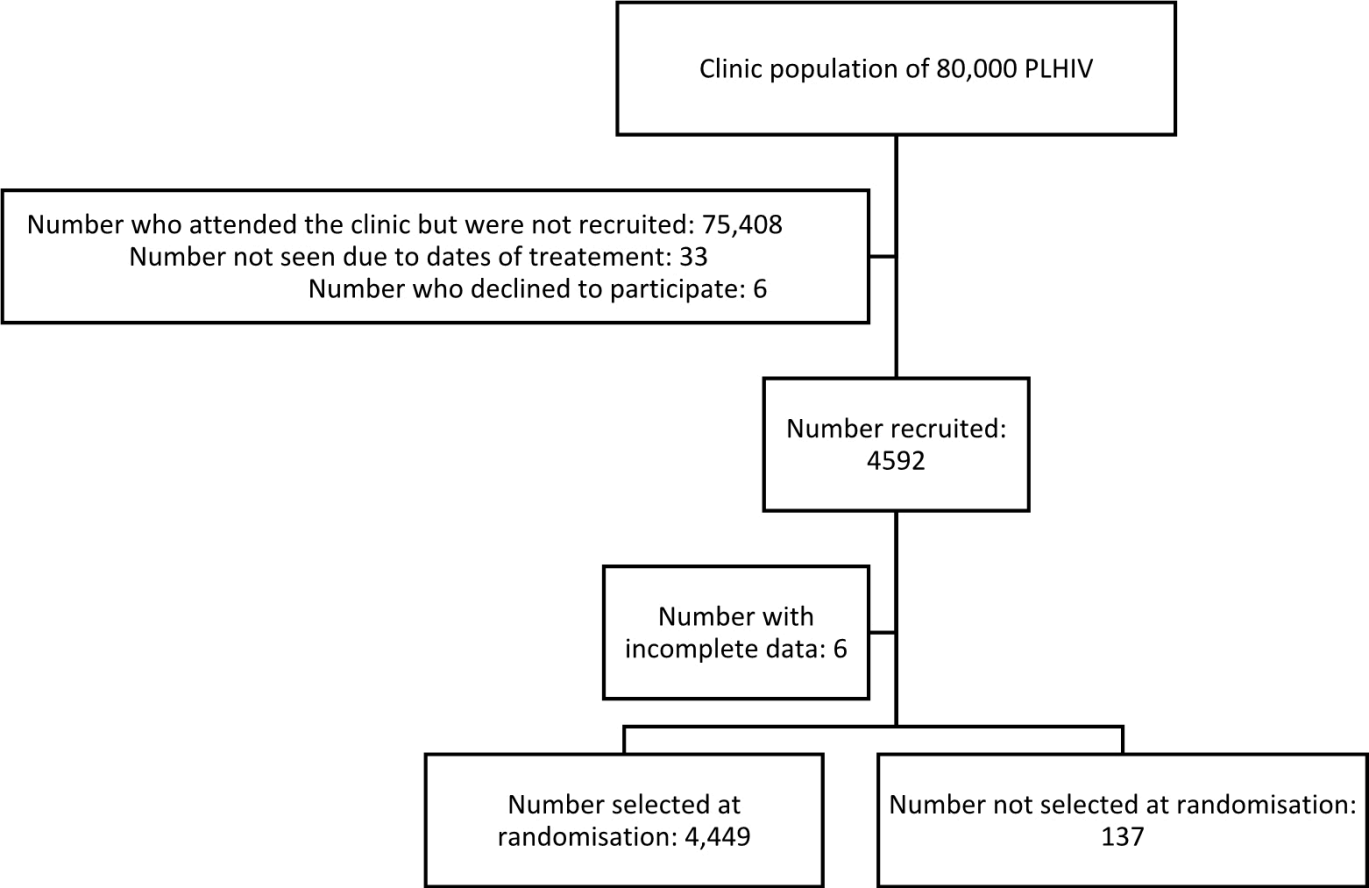
Participant flow diagram

Participant recruitment for the primary study was stopped early at 4,565 participants after recalculation of the sample size showed that the parent study objectives had been achieved. From this a random sample of the required 4,449 records was selected as shown in the participant flow diagram (see figure 1), which also provides a summary the participant recruitment details. There were 3,103/4,449 (69.7%) female participants for this study. The mean age was 44.3 years (SD 10.1 years) with a range of 18 to 89 years. Table 1 provides additional descriptive information on the selected study participants.

### Oral care practices

It is important to note that about 43.9% (1,951/4,449) of the participants in this study on people living with HIV had never been seen by a health worker prior to this study for a disease related to their mouth. Of the remainder that had seen a health worker for a disease related to their mouth, only 16.5% (736/4,449) had done so in the 12 months prior to being recruited into this study. Out of the 2,499/4,449 (56.2%) that had been seen by a health worker for a disease related to their mouth the majority (1,200/2,499, 48%) visits were treatment related, 45,3% (1,132/2499) visits were due to pain with teeth or gums or in the mouth, 5.2% (131/2,499) were for consultation or advice, 1.2% (29/2499) followed having wounds in the mouth and 0.3% (7/2499) could not remember why they had gone to see the health workers. Table 2 provides additional information on the frequency of the visits in the 12 months prior to recruitment into the study and other participants oral care practices. In this table it is important to note that only 6.5% (290/4449) of the participants in this study had seen a dentist for the recommended two or more times over a span of 12-months.

### Periodontal health status of PLHIV:

#### Gingival health score:

As shown in table 1 above, 65.4% of the study participants had bleeding. Additionally, we found 1,533/4,443 (34.5%) with no bleeding at all, 337/4,443 (7.6%) with bleeding in one sextant, 411/4,443 (9.3%) with bleeding in two sextants, 448/4,443 (10.1%) with bleeding in three sextants, 448/4,443 (10.1%) with bleeding in four sextants, 415/4,443 (9.3%) with bleeding in five sextants, and 851/4,443 (19.2%) with bleeding in all six sextants. There were 6/4,449 (0.13%), individuals with absent index/selected or the next teeth who were excluded from the above analysis. Multilevel regression, controlling for the site of the tooth and the participant, was used to model bleeding on probing as the dependant (score 0 which was for “no bleeding”, as the reference). Controlling for the other factors, female participants were significantly more likely to have bleeding on probing compared to male participants (adjusted Odds ratio: 1.49, 95% CI 1.19 to 1.86). The more teeth a participant had the more likely they were to have observed bleeding on probing. This was significant (adjusted odds ratio 1.04, 95% CI 1.01 to 1.07). Also, the use of any form of tobacco was associated with significantly increased odds of bleeding on probing (adjusted odds ratio 1.62, 95% CI 1.09 to 2.41). It was also noted that the presence of pockets (adjusted odd ratio 0.22, 95% CI 0.16 to 0.28) and the presence of clinical attachment loss (adjusted odds ratio 0.40, 95% CI 0.35 to 0.45) were both associated with significant reduction in odds for bleeding on probing.

#### Periodontal Pocket Depth:

As shown in Table 1, there were 3,735/4,439 (84.1%) participants with a score of 0 (absence of condition/ 0–3mm) for all the six assessments of the selected sentinel/Ramfjord teeth. Looking at each of the six individual pocket score assessments per tooth examined, we found, 347,755 (93.3%) of the tooth sites examined with a score of 0 (absence of condition/ 0–3mm), 22,673 (6.1%) of the tooth sites examined with a score of 1 (pocket depth of 4–5mm) and 2,154 (0.6%) of the tooth sites examined with a score of 2 (pocket depth of 6-mm or more). On modelling, no significant associations were observed between periodontal pocket depth and age, gender, number of available teeth, alcohol or tobacco use and duration of time on HAART.

#### Clinical attachment loss:

As shown in Table 1, there were 2,293/4,440 (51.6%) with no clinical attachment loss (score of 0 or 0–3 mm). Table 3 above, shows the number of participants for each duration on HAART and the corresponding score for clinical attachment loss. Multilevel regression as summarised in Table 4, controlling for the site of the tooth and the participant revealed the following, from a model with the dependant as clinical attachment presence or absence. Controlling for the other factors in the model, the odds for finding presence of clinical attachment loss increased with increasing age. In Table 4 note the significant increase in the odds for clinical attachment loss from the youngest to the oldest age group relative to participants under the age of 30-years. In the same table note that the Female participants were significantly less likely to have clinical attachment loss compared with male participants. In this table we see that there were almost twice as many participants with higher scores of clinical attachment loss in the one year but less than two-years’ period compared to the reference period of less than six months on HAART group controlling for all other factors in the model. Controlling for all the other factors in the model note the threefold increase in clinical attachment loss in participants that had been on HAART for more than one year, but less than two years compared individuals who were on HAART for less than 6 months. This was the only period with a significant odds ratio (adjusted odds ratio: 2.47, 95% CI 1.04 to 5.91). Also, in Table 4 also note that presence of pockets was associated with an almost three-fold increase in clinical attachment loss while bleeding on probing was associated with a 70% reduction in clinical attachment loss.

## Discussion

We set out to describe the periodontal health of a large cohort of Ugandans living with HIV. For the measures of periodontal health (bleeding on probing, probing pocket depth and clinical attachment loss), the major observations were that about 66% of the participants had bleeding on probing at one or more of the examined sites/tooth surfaces. The odds for bleeding on probing were significantly higher for female participants, and higher in individuals who reported tobacco use. This corresponds to observations from previous studies, that report an increased risk of bleeding on probing for the female gender [29]. For tobacco use, we observed 60% increase in the odds for bleeding, which is the reverse of what has been reported in literature [30]. This may have been due to the presence of plaque that has been reported to have an almost three-fold increase in bleeding on probing for non-smokers [30]. For this study we did not collect any plaque related data though using the information in table 2 on the reported oral hygiene data support this assertion.

For probing pocket depth, we noted that no significant associations were found with age, gender, number of teeth present, alcohol or tobacco use and duration of time on HAART. It is possible that the events leading to the development of pockets are local in nature [31]. This is in part supported by the observation that bleeding on probing is reduced significantly in the presence of pockets for this population. It is important to note that most of the participants in this study (84%) did not have any pockets at any of the examined sites. This and the previously mentioned bleeding on probing points to the presence of a mild reversible form of periodontal disease also known as gingivitis. For some of the participants, this gingivitis may now be presenting on top of a previously healed episode of periodontitis. This is possible because periodontal health, by definition, encompasses four distinct states of health [32]. Once periodontal disease progress past the first two states of the pristine periodontal health, characterized by a structurally sound and uninflamed periodontium or the second which is a well-maintained clinical periodontal health, that has structurally and clinically intact periodontium, we have periodontitis that may be in an active state, stable state, or remission, remains irreversible.

### Clinical attachment loss

Looking at clinical attachment loss we noted that slightly under half of our participants (48.2%) had moderate to severe clinical attachment loss. The extent of clinical attachment loss in this population is similar to that seen in a study from Rwanda, another country in East Africa, where 45% of the participants with HIV had clinical attachment loss [33]. Both are higher than the observed prevalence of clinical attachment loss of 34.7% in a similar ART-experienced population in Cameroon that is in western Africa [26]. This higher level of clinical attachment loss for our study population is in tandem with the general population as per global reports showing a high prevalence of periodontal disease in east African region [34]. This makes the relatively high finding of clinical attachment loss, a defining symptom of periodontitis, a point of concern for this cohort. On the other hand, our observed prevalence of clinical attachment loss was similar that from another study in India that found 49.1% of participant living with HIV had clinical attachment loss [35]. Therefore, PLHIV may need special attention for their periodontal health even when they are complaint with their HAART medication. [35].

Our data on increasing clinical attachment loss, a characteristic feature of periodontitis, with age and on HAART treatment may be explained in part by the following observations. From the results in Table 3, the odds for clinical attachment loss increase with time. This is captured in the variables with data on both the age groups and the time on HAART. In the severe forms of periodontitis there is apical migration of the epithelial attachment accompanied by loss of connective tissue and alveolar bone [10]. These changes in the structures that surround the teeth leading to pockets and eventual bone loss are mediated by the interaction between pathogens like *Porphyromonas gingivalis* [36], and the host immune-inflammatory response [37, 38]. Finally, it is also important to note that oncethe destruction of the periodontium begins, it is currently considered as irreversible and permanent, which makes structural and functional maintenance of the periodontium important [39]. Chronic periodontitis affects 66% of HIV-patients [39]. Importantly, the HIV infection, even when controlled, alters the human microbiome, and contributes to a chronic low-grade inflammatory state [40].

The presence of pockets and clinical attachment loss (CAL) is typically associated with a higher likelihood of bleeding gums (gingival bleeding). However, in these patients on Highly Active Antiretroviral Therapy (HAART), the opposite is observed, with lower odds of bleeding gums despite the presence of these periodontal disease markers. One possible explanation for this unexpected finding is the immunosuppressive state of HIV patients, which may mask the typical inflammatory response that leads to bleeding gums. HAART therapy suppresses the viral load, allowing the immune system to recover, but this recovery may take time. Within the first 6–12 months of commencing HIV treatment, the immune system is still rebuilding, and the typical inflammatory response to periodontal disease may be dampened. As a result, bleeding gums may be less likely to occur, despite the presence of periodontal pockets and CAL. Another possible explanation is the effect of HAART on the oral microbiome. HAART may alter the balance of oral bacteria, reducing the presence of periodontal pathogens and leading to a decrease in gingival bleeding. It’s important to note that this is a hypothetical explanation and further research is needed to fully understand the relationship between HAART, periodontal disease, and gingival bleeding in HIV patients [41, 42].

### Limitations of the study

This study employed a cross-sectional design, precluding the observation of cyclical changes in periodontal health over time. While evidence of such variations was identified amongst participants with diverse periodontal conditions, future investigations should utilize longitudinal designs to capture prospective data and control for individual-level factors. Furthermore, the study acknowledged the ongoing evolution of periodontal health assessment methods and the heterogeneity of methodologies employed in prior research [43]. The adoption of the 2013 WHO-modified Community Periodontal Index (WHO-mCPI) was deemed appropriate due to its suitability for epidemiological studies and its ability to facilitate comparisons with data from other populations [27]. However, the focus on individuals living with HIV limited the generalizability of findings to the HIV-negative population. It is recognized that periodontal health outcomes can be influenced by various factors beyond HIV status, including genetics and the oral microbiome. Therefore, cautious interpretation of the study’s findings is warranted.

## Conclusions

This study investigated the periodontal health of a large cohort of Ugandans living with HIV. We observed a high prevalence of bleeding on probing (66%) and moderate to severe attachment loss (48.2%). While factors like age, gender, and tobacco use influenced bleeding on probing, clinical attachment loss increased with both age and duration of HAART treatment. These findings suggest a potential link between immune reconstitution and increased attachment loss, requiring further investigation with longitudinal studies. Our study highlights the need for special attention to the periodontal health of PLHIV, even with successful HAART treatment. Future studies should employ longitudinal designs to capture temporal changes and utilize standardized measures for better generalizability. Additionally, including a control group without HIV would strengthen the understanding of how HIV infection itself contributes to periodontal health. Despite limitations, this study provides valuable epidemiological data on the periodontal health of Ugandans living with HIV. This information can inform the development of targeted interventions to improve their oral health outcomes.

## Figures and Tables

**Table 1:** Descriptive statistics for the study population

Observation	Overall (N = 4449)
Age (years)	
Mean (SD)	44.3 (10.1)
Median [Min to Max]	44.0 [18.0 to 89.0]
Gender	
Male	1346 (30.3%)
Female	3103 (69.7%)
Available teeth	
Mean (SD)	28.3 (3.83)
Median [Min to Max]	29.0 [1.00 to 33.0]
Missing	6 (0.1%)
Tobacco use	
Never	4148 (93.2%)
User	301 (6.8%)
Alcohol consumption	
Never	3187 (71.6%)
Used in last month	1262 (28.4%)
How long have you been on ARVs	
Less than 6 months	75 (1.7%)
6–12 months	86 (1.9%)
More than 1 year but less than 2 years	102 (2.3%)
Two years or more but less than 5 years	296 (6.7%)
5 years or more	3890 (87.4%)
Gingivitis	
bleeding	2910 (65.4%)
Normal	1533 (34.5%)
Missing	6 (0.1%)
Highest Pocket depth score	
score_0	3735 (84.0%)
score_1	622 (14.0%)
score_2	82 (1.8%)
Missing	10 (0.2%)
Highest Clinical attachment loss score	
score_0	2293 (51.5%)
score_1	1473 (33.1%)
score_2	566 (12.7%)
score_3	78 (1.8%)
score_4	30 (0.7%)
Missing	9 (0.2%)
Level of Education	
Postgraduate Degree	13 (0.3%)
College/University completed	236 (5.3%)
A level completed/ Tertiary institution after O level (S6)	362 (8.1%)
Secondary school (O level) completed (S4-S5)	626 (14.1%)
Primary school completed (P7-S3)	1481 (33.3%)
Less than primary school (P1-P6)	1454 (32.7%)
No formal schooling (No Education)	277 (6.2%)
Occupation	
No employment/stay home	574 (12.9%)
Unskilled labor (Shopkeeper/Potter/Maid)	1119 (25.2%)
Agriculture (Peasant/Subsistence Farmers)	510 (11.5%)
Self-employed/business	1345 (30.2%)
Skilled labor (Carpenters/ Tailors/ Mechanics)	449 (10.1%)
Sales and services/clerical	105 (2.4%)
Student	29 (0.7%)
Professional/managerial	253 (5.7%)
Other	65 (1.5%)

**Table 2:** Summary of participants reported oral hygiene practices.

Observation	Overall (N = 4449)
1. How often did you go to the dentist during the past 12 months?	
Once	472 (10.6%)
Twice	209 (4.7%)
Three times	43 (1.0%)
Four times	13 (0.3%)
More than four times	25 (0.6%)
I had no visit to dentist during the past 12 months	3103 (69.7%)
I have never received dental care/visited a dentist	578 (13.0%)
I don’t know or don’t remember	6 (0.1%)
2. How often do you clean your mouth?	
Never	3 (0.1%)
Several times a month (2–3 times)	5 (0.1%)
Once a week	12 (0.3%)
Several times a week (2–6 times)	16 (0.4%)
Once a day	1636 (36.8%)
2 or more times a day	2777 (62.4%)
Toothbrush	
Yes	4380 (98.4%)
No	69 (1.6%)
Wooden Toothpicks	
Yes	2229 (50.1%)
No	2220 (49.9%)
Plastic toothpicks?	
Yes	139 (3.1%)
No	4310 (96.9%)
Thread (dental floss)	
Yes	157 (3.5%)
No	4292 (96.5%)
Chewstick/Miswak	
Yes	795 (17.9%)
No	3654 (82.1%)
3. Do you use toothpaste to clean your teeth?	
Yes	4172 (93.8%)
No	277 (6.2%)
Soap	
Yes	886 (19.9%)
No	3563 (80.1%)
Salt	
Yes	1206 (27.1%)
No	3243 (72.9%)
Local herbs/Herbal Concoctions	
Yes	126 (2.8%)
No	4323 (97.2%)
Ash	
Yes	660 (14.8%)
No	3789 (85.2%)
Charcoal	
Yes	694 (15.6%)
No	3755 (84.4%)
Herbal Mouthwashes	
Yes	181 (4.1%)
No	4268 (95.9%)

**Table 3:** Duration on HAART and highest scores of clinical attachment loss

	Clinical attachment loss highest score (column percent)					
How long have you been on ARVs	score_0.	score_1.	score_2	score_3.	score_4.	Total.
Less than 6 months	47 (2.0%)	23 (1.6%)	5 (0.9%)	0 (0.0%)	0 (0.0%)	75 (1.7%)
6–12 months	51 (2.2%)	25 (1.7%)	8 (1.4%)	1 (1.3%)	1 (3.3%)	86 (1.9%)
More than 1 year but less than 2 years	51 (2.2%)	32 (2.2%)	17 (3.0%)	1 (1.3%)	1 (3.3%)	102 (2.3%)
2 years or more but less than 5 years	168 (7.3%)	98 (6.7%)	21 (3.7%)	5 (6.4%)	4 (13.3%)	296 (6.7%)
5 years or more	1976 (86.2%)	1295 (87.9%)	515 (91.0%)	71 (91.0%)	24 (80.0%)	3881 (87.4%)
Total	2293	1473	556	78	30	4440

**Table 4:** showing model for clinical attachment loss presence in people living with HIV

term	Adj.OR	statistic	p.value	conf.low	conf.high
(Intercept)	2.07	1.53	0.13	0.81	5.29
Age group (less than 20 years)	Ref	-	-	-	-
Age group (30 to 40 years)	2.26	4.61	0.00	1.60	3.20
Age group (50 to 60 years)	3.65	6.91	0.00	2.53	5.27
Age group (Elderly)	6.89	4.43	0.00	2.94	16.18
Gender (Female)	0.50	−7.79	0.00	0.42	0.59
Used alcohol in last month	0.96	−0.41	0.68	0.80	1.15
Is a tobacco user	1.28	1.50	0.13	0.93	1.76
Been on ARVs less than 6 months	Ref	-	-	-	-
On ARVs for 6–12 months	1.15	0.32	0.75	0.48	2.75
On ARVs more than 1 year but less than 2 years	2.69	2.35	0.02	1.18	6.14
On ARVs for 2 years or more but less than 5 years	1.55	1.20	0.23	0.76	3.16
On ARVs for 5 years or more	1.57	1.35	0.18	0.82	3.01
Gingival bleeding on probing	0.36	−17.45	0.00	0.32	0.40
Gingival pockets present	2.48	8.49	0.00	2.01	3.06
Number of teeth found	0.88	−11.57	0.00	0.86	0.90
